# Discovery of Novel Pain Regulators Through Integration of Cross‐Species High‐Throughput Data

**DOI:** 10.1111/cns.70255

**Published:** 2025-02-09

**Authors:** Ying Chen, Akhilesh K. Bajpai, Nan Li, Jiahui Xiang, Angelina Wang, Qingqing Gu, Junpu Ruan, Ran Zhang, Gang Chen, Lu Lu

**Affiliations:** ^1^ Department of Histology and Embryology, Medical College Nantong University Nantong Jiangsu China; ^2^ Department of Genetics, Genomics and Informatics University of Tennessee Health Science Center Memphis Tennessee USA; ^3^ Medical College Nantong University Nantong Jiangsu China; ^4^ Department of Cardiology Affiliated Hospital of Nantong University Jiangsu China; ^5^ Department of Anesthesiology Affiliated Hospital of Nantong University Jiangsu Province China

**Keywords:** BXD mice, cross‐species integrated approach, pain, RNA sequencing, spared nerve injury

## Abstract

**Aims:**

Chronic pain is an impeding condition that affects day‐to‐day life and poses a substantial economic burden, surpassing many other health conditions. This study employs a cross‐species integrated approach to uncover novel pain mediators/regulators.

**Methods:**

We used weighted gene coexpression network analysis to identify pain‐enriched gene module. Functional analysis and protein‐protein interaction (PPI) network analysis of the module genes were conducted. RNA sequencing compared pain model and control mice. PheWAS was performed to link genes to pain‐related GWAS traits. Finally, candidates were prioritized based on node degree, differential expression, GWAS associations, and phenotype correlations.

**Results:**

A gene module significantly over‐enriched with the pain reference set was identified (referred to as “pain module”). Analysis revealed 141 pain module genes interacting with 46 pain reference genes in the PPI network, which included 88 differentially expressed genes. PheWAS analysis linked 53 of these genes to pain‐related GWAS traits. Expression correlation analysis identified Vdac1, Add2, Syt2, and Syt4 as significantly correlated with pain phenotypes across eight brain regions. NCAM1, VAMP2, SYT2, ADD2, and KCND3 were identified as top pain response/regulator genes.

**Conclusion:**

The identified genes and molecular mechanisms may enhance understanding of pain pathways and contribute to better drug target identification.

## Graphical abstract

Cross‐species analysis was used to identify novel pain mediators based on human DRG expression profiles and RNA‐seq data from a mouse model of neuropathic pain injury.

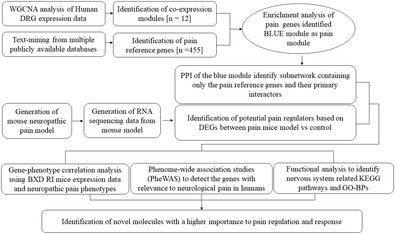

## 1 Introduction

Pain is categorized into three major types: nociceptive, inflammatory, or neuropathic [1, 2]. Nociceptive pain plays an important role in avoidance of injury and often subsides following the completion of healing process. It arises due to activation of a specific type of sensory neuron called as nociceptor primarily by external stimulants, such as temperature, mechanical stimulation, and chemical irritants [3]. Inflammatory pain, on the other hand is caused by local inflammatory response and pain hypersensitivity as a result of tissue injury [4]. Neuropathic pain, however, is different from nociceptive and inflammatory pain because the underlying cause of it is nerve lesions that alter the activity of nerve fibers. Although peripheral nervous system (PNS) is mainly responsible for the origin of neuropathic pain, it may also begin in the central nervous system (CNS) [5].

Chronic pain (i.e., pain lasting ≥ 3 months) is an encumbering condition that affects day‐to‐day life. According to the recent estimates, in the United States alone, chronic pain affects ~21% of adults (~52 million individuals) [6] and has been linked with depression [7], neurodevelopmental disorders [8], substance use and misuse [9], and higher suicidal risks [10]. The prevalence of high‐impact chronic pain in 2021 was estimated to be 6.9%, which was lower than that of 8% in 2016 [11]. However, when the age‐adjusted prevalence of chronic pain in 2021 was calculated, it was close to the goal set by the Healthy People 2030 objective with 6.4% (https://health.gov/healthypeople/objectives‐and‐data/browse‐objectives/chronic‐pain) [6]. Pain is not only a health burden but also a financial burden. The economic burden of pain has been estimated to be higher than most other health conditions, including heart diseases and cancer treatments, with an annual cost of ~$600 billion in 2010, which is composed of direct healthcare expenditure, days/h of work missed, and lower wages [1, 12].

Conventional oral analgesics are always the first choice of treatment for pain management. During pain treatment, the physician usually follows the steps on the World Health Organization (WHO) analgesic ladder [13]. The first step on the ladder is non‐opioid analgesics followed by weak opioids, such as codeine as the second step. The opioid drugs mimic the effects of endorphins, the naturally occurring pain reducing chemicals. However, long‐term usage (more than 6 months) of codeine has been shown to increase the risk of serious thrombotic cardiovascular events in older patients [14, 15]. The third step on the WHO ladder is using the more potent strong opioids; however, they have more severe side effects [15]. Decades of research has helped us in gaining some insights into the molecular mechanisms underlying pain response and regulation, and identification of genes linked to pain [1, 16, 17]. Although several preclinical models for studying pain have been developed, there are only a few biomarkers that have broad utility in clinical practice [18, 19]. This complexity could be due to the involvement of multiple mechanisms and cell types in pain response/regulation, which is further aggravated by the spectrum of factors that influence pain, such as psychological, emotional, and environmental aspects. To overcome the hurdles associated with the treatment and management of pain, there is a need to focus on using better animal models that closely mimic the human system. In addition, it is of utmost importance to replicate the findings from animal models to patients through an integrated cross‐species approach.

The mouse genetic reference population (GRP), such as the BXD is a suitable animal model for studying the pain response and regulation. These strains have been derived from the crosses between C57BL/6J (B6) and DBA/2 J (D2) mice for more than 20 consecutive generations [20, 21]. Currently, there are more than 150 BXD stains available with hundreds of omics datasets generated from various tissues including different brain regions and thousands of phenome datasets, including several pain phenotypes [21]. All these data can be accessed and analyzed through our GeneNetwork portal (https://genenetwork.org/). As the BXD GRP is a fully‐inbred population, each strain can be replicated in large numbers, facilitating precise mapping of complex traits [21]. The BXD population has been used in past decades to uncover the molecular mechanisms and identify candidate genes in various physiological and pathological conditions [22, 23, 24, 25, 26], making it an invaluable resource for systems genetics studies.

The advancement in next‐generation high‐throughput sequencing has emerged as a boon for the scientific filed of discovery. The sequencing of mRNA at a large scale has helped us in understanding the novel cellular and molecular mechanisms that seemed far‐fetched a decade ago [27, 28]. RNA sequencing has been extensively used in pathology research, including for unraveling the aspects linked to pain response and regulation [29, 30, 31]. The availability of large‐scale data fuelled the development of a number of algorithms and approaches for facilitating the analysis and interpretation of such data. Weighted Gene Co‐Expression Network Analysis (WGCNA) is one such approach that calculates the weighted adjacency matrix using the expression pattern of genes and results in coexpressed gene clusters [32]. The WGCNA method has been widely used in identifying clusters of genes that eventually aid in the discovery of biomarkers or candidate genes for various conditions including pain physiology [33, 34, 35].

In the current study, first, we used microarray gene expression profiles of > 200 human dorsal root ganglion (DRG) samples to identify co‐expressed gene modules and then selected a module that was significantly over‐enriched for pain reference genes. Functional analysis of the selected module (pain module) indicated enrichment of several pathways and ontologies linked to pain response and regulation. Protein–protein interaction network of the pain module led to the identification of potential pain regulator/response genes. Furthermore, we performed RNA sequencing of pain model (spared nerve injury (SNI) mice) versus control mice to validate the differential expression of the network genes. Finally, the pain regulators were identified based on genome wide association analysis in humans, correlation with pain phenotypes in BXD mice, node degree in the protein interaction network and strong differential expression between pain model and control mice. Our study used a cross‐species integrative approach to identify genes strongly associated with pain response and regulation.

## 2 Materials and Methods

### 2.1 Animals

Male ICR mice, 6–8 weeks of age, were obtained from the Experimental Animal Center of Nantong University, China (animal license number: SCXK (Su) 2014‐0001 and SYXK (Su) 2012‐0031) and were used for deep sequencing. Male mice are common research subjects in pain modeling, considering that the effect of pain response in female mice may be affected by the hormonal cycle [36, 37]. Hence, we used male mice to construct the model of neuropathic pain in this study. All animal experiments were conducted in accordance with the Animal Research Committee of Nantong University Medical School (permission no. S20220303‐005). Three mice were used for each of the SNI and control groups.

### 2.2 Neuropathic Pain Model

SNI was used to induce neuropathic pain [38]. Mice were placed in a prone position and operated with isoflurane using anesthesia machine (RWD Life Science, Jiangsu, China). During the surgery, the sciatic nerve was completely exposed to the three bifurcates. The proximal tibial nerve and common peroneal nerve were ligated with 6.0 silk thread, the distal end of the ligation was cut, and the sural nerve was preserved. The control group underwent the same surgeries described above but without nerve ligation. The dorsal horn of lumbar (L4–L6) spinal cord was collected on the 14th day after SNI for RNA‐seq.

### 2.3 RNA Isolation, Sequencing, and Data Analysis

Total RNA from spinal cord tissue was extracted using Trizol reagent kit (Invitrogen, Carlsbad, CA, USA) according to the manufacturer's protocol. RNA quality was assessed on an Agilent 2100 Bioanalyzer (Agilent Technologies, Palo Alto, CA, USA) and checked using RNase‐free agarose gel electrophoresis. After total RNA was extracted, mRNA was enriched by Oligo(dT) beads. Then the enriched mRNA was fragmented into short fragments using fragmentation buffer and reverse transcribed into cDNA by using NEBNext Ultra RNA Library Prep Kit for Illumina (NEB #7530, New England Biolabs, Ipswich, MA, USA). The purified double‐stranded cDNA fragments were end repaired, “A” base added, and ligated to Illumina sequencing adapters. The ligation reaction was purified with the AMPure XP Beads (1.0X). Ligated fragments were subjected to size selection by agarose gel electrophoresis and polymerase chain reaction (PCR) amplified. The resulting cDNA library was sequenced using Illumina Novaseq6000 by Gene Denovo Biotechnology Co. (Guangzhou, China). An average of ~50 million 150 bp paired‐end reads was sequenced per sample.

The raw reads were filtered by fastp (version 0.18.0) [39] to remove the low‐quality sequences using the following parameters: (1) removing reads containing adapters; (2) removing reads containing more than 10% of unknown nucleotides (N); and (3) removing low quality reads containing more than 50% of low quality (*Q*‐value ≤ 20) bases. The clean reads were used for alignment and gene abundance calculation. An index of the reference genome (mm10) was built, and paired‐end clean reads were mapped to the reference genome using HISAT2. 2.4 [40] with “‐rna‐strandness RF” and other parameters set as a default. The mapped reads of each sample were assembled by using StringTie v1.3.1 [41, 42] in a reference‐based approach. The fragment per kilobase of transcript per million mapped reads (FPKM) value was calculated to quantify its expression abundance and variations, using RSEM [43] software. Differential expression analysis was performed by edgeR [44] between pain and control group. The genes with Benjamini‐Hochberg corrected [45] false discovery rate (FDR) below 0.05 and absolute fold change ≥ 1.5 were considered differentially expressed.

### 2.4 Weighted Gene Co‐Expression Network Analysis

**FIGURE 1:**
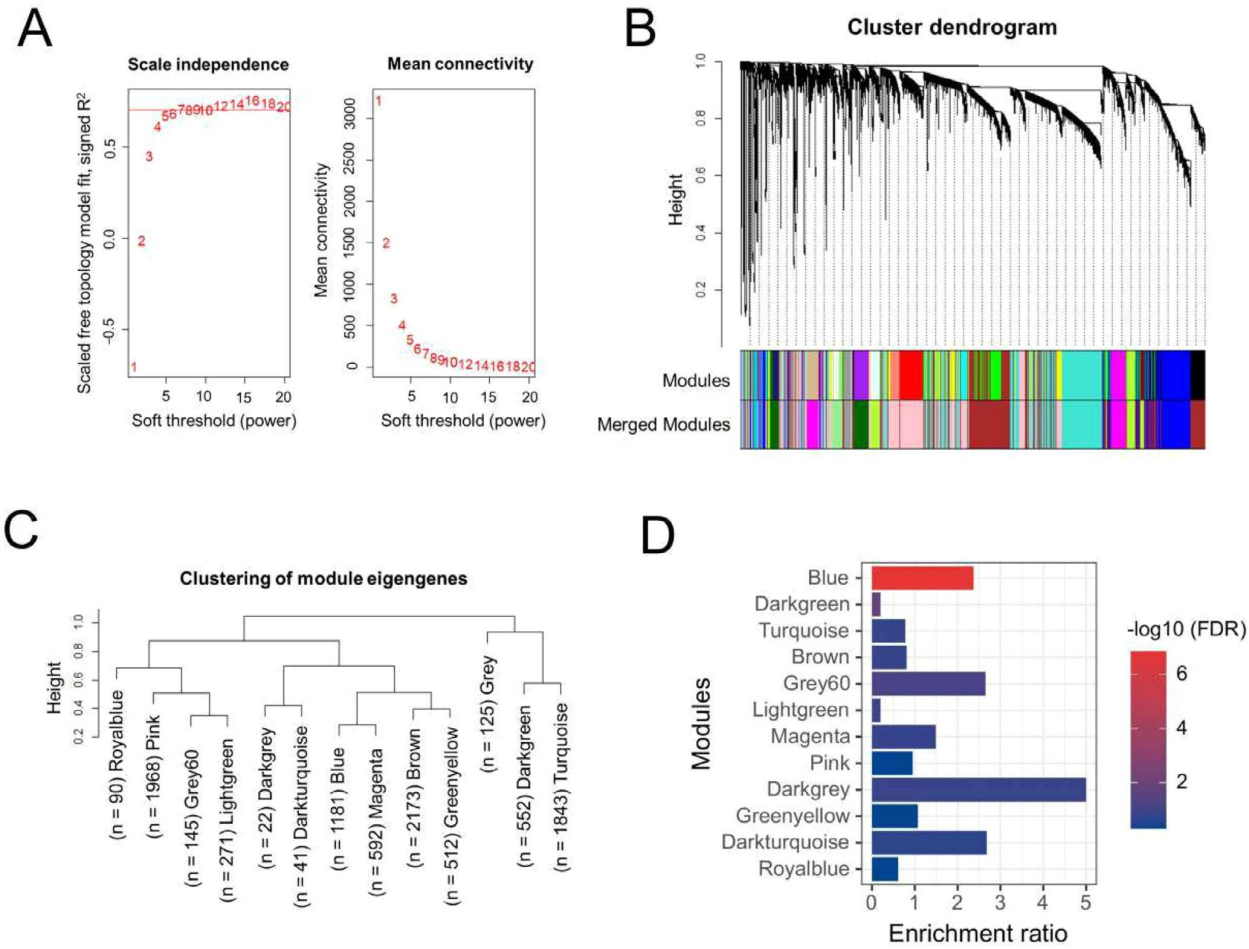
WGCNA analysis and identification of pain module. (A) Soft‐thresholding index *R* ^2^ or mean connectivity (*y*‐axis) as a function of different *β* thresholds (*x*‐axis). A *β* of 8 was selected for constructing the coexpression network. (B) Gene dendrogram obtained by clustering the dissimilarity values based on consensus Topological Overlap. The color rows show the preliminary (Modules) and the merged module assignments (Merged modules). A total of 25 preliminary modules were detected by clustering the top 10,000 genes expressed across 206 human dorsal root ganglion samples. Merging the modules with a distance threshold of 0.25 resulted in 13 modules. (C) Hierarchical clustering of the 13 modules. Numbers indicated next to the module names represent the total number of genes in each module. Gray module contains the unassigned genes and was not considered for further analysis. (D) Barplot representing the enrichment of pain reference genes in each module. Blue module was found to be significantly over‐enriched for pain reference genes and was considered as the “pain module”.

The WGCNA is a method that is used to construct the co‐expression network of genes and explore the associations between gene expression patterns. We used WGCNA package [32] in R to construct a co‐expression network and identify significant modules using the human DRG gene expression dataset, GSE77968 that contains 214 DRG samples obtained from brain‐dead human subjects [46]. Briefly, the samples were profiled on Affymetrix Human Transcriptome Array 2.0 platforms and were normalized using Robust Multi‐Array Average (RMA) algorithm by considering all the samples simultaneously; additional details on sample processing and analysis can be obtained from the original study [46]. The samples were first clustered based on normalized intensity to detect and remove the outliers using *hclust* function and average linkage method, leaving a total of 206 samples that were finally used for the network analysis. Top 10,000 genes based on the mean expression across 206 samples were selected for the network construction. Briefly, the weighted adjacency matrix was constructed using the soft‐thresholding power (*β*) of 8 to attain scale‐free topology. The soft‐thresholding is a value used to power the correlation of the genes to that threshold. Here, we assume that by increasing the correlation to a power will reduce the noise of the correlations in the adjacency matrix. To pick up the power, we used the *pickSoftThreshold* function, which calculates if the network resembles a scale‐free graph for each power. The soft‐thresholding power of 8 in our case produced a higher similarity with a scale‐free network (as shown in Figure 1A), hence it was considered further. The adjacency matrix was converted to topologically overlapping matrix and then to the dissimilarity matrix. The dissimilarity matrix was used to hierarchically cluster the genes, which were then assigned to different coexpressed modules. A minimum module size was set to 50 and dynamic tree cutting was used to identify the modules. Furthermore, to quantify co‐expression similarity of entire modules, we calculated their eigengenes and clustered them on their correlation. A distance threshold of 0.25 (similarity of 75%) was used to merge similar modules. The gray module contains the unassigned genes; hence it was excluded from further analysis.

### 2.5 Generation of Pain Reference Gene Set and Identification of Pain Module

The pain‐related genes were collected from multiple publicly available resources using keywords, such as “pain”, “neuropathic pain”, and “neuralgia”. These keywords were searched in the following databases/repositories: DISEASES database [47] (https://diseases.jensenlab.org/), GeneCards [48] (https://www.genecards.org/), Rat Genome Database [49] (RGD: https://rgd.mcw.edu/), and GWAS Catalog [50] (https://www.ebi.ac.uk/gwas/). The pain‐related genes based on automatic text‐mining were retrieved from the DISEASES database. Furthermore, genes with a relevance score ≥ 3 were considered from the GeneCards database. The relevance score calculated by the GeneCards database is based on term frequency/inverse document frequency (additional details on the scoring can be found here: https://www.genecards.org/Guide/Search#relevance). In brief, the higher the relevance score, the stronger is the association between a gene and a term. Finally, a comprehensive list of pain‐related genes was derived by combining the above sets and then removing duplicates. This final set obtained is henceforth referred to as the “pain‐reference geneset” (File S1).

To identify the pain‐specific module among the modules identified based on WGCNA analysis, we performed an enrichment analysis for each coexpression module using the “pain reference geneset”. The statistical significance of the enrichment was tested using hypergeometric test and Benjamini‐Hochberg correction [45] was used for controlling the FDR. An FDR corrected *p* < 0.05 was used as a significance threshold.

### 2.6 Protein–Protein Interaction (PPI) Network Construction and Analysis

The PPI network among the selected proteins was constructed by retrieving the protein interactions from the search tool for retrieval of interacting genes (STRING) database (https://string‐db.org/) [51, 52]. The interactions in STRING database are based on multiple evidence, including text mining, experiments, co‐expression, neighborhood, gene fusion, and co‐occurrence. We used a high‐threshold score of 0.7 to obtain a reliable set of interactions among the proteins. The interactions were then uploaded onto Cytoscape v3.9.1 platform [53] for further analysis and visualization. CytoHubba v0.1 [54] application plugin was used to analyze the PPI network using 11 topological analysis methods.

### 2.7 Brain Expression Data From BXD Mice

The gene expression data corresponding to different brain regions of BXD mice generated by us and our collaborators were obtained from our GeneNetwork portal (https://genenetwork.org/) [55]. The expression data from the following brain regions were used in the current study: amygdala, hypothalamus, cerebellum, nuclear accumbens (NAc), prefrontal cortex (PFC), neocortex, ventral tegmental area (VTA), and hippocampus. A total of 56 BXD strains were analyzed to generate the amygdala expression dataset [GN323: INIA Amygdala Cohort Affy MoGene 1.0 ST (Mar11) RMA]. The hypothalamus expression data were generated using 48 BXD strains [GN281: INIA Hypothalamus Affy MoGene 1.0 ST (Nov10)]. Cerebellum expression data were generated from 28 BXD strains [GN72: GE‐NIAAA Cerebellum mRNA M430v2 (May05) RMA]. The expression data for NAc were from 34 strains [GN156: VCU BXD NAc Sal M430 2.0 (Oct07) RMA]. A total of 27 BXD strains were used for generating the PFC expression data [GN135: VCU BXD PFC Sal M430 2.0 (Dec06) RMA]. The neocortex expression data were generated from 52 BXD strains [GN284: HQF_BXD_Neocortex_ILM6v1.1_Dec10v2_RankInv]. The expression data from VTA were generated from 35 BXD strains [GN228: VCU BXD VTA Sal M430 2.0 (Jun09) RMA], whereas hippocampal gene expression dataset was obtained from 69 BXD strains [GN110: Hippocampus Consortium M430v2 (Jun06) RMA]. All expression datasets were based on the microarray platforms and contained data from both the parental strains (C57BL/6J and DBA/2J) as well. Additional details on the generation and processing of the expression data can be found in our GeneNetwork database (http://genenetwork.org/).

### 2.8 Gene‐Phenotype Correlation

For gene‐phenotype correlation analysis, we correlated the mRNA levels of the genes in different brain regions with neuropathic pain phenotype measurements generated by us and our collaborators. For the current analysis, we used a total of 31 pain phenotypes that were primarily related to thermal nociception, chemical nociception and mechanical nociception in BXD mice (File S2). These phenotypes can be accessed through our GeneNetwork database (http://genenetwork.org/). Pearson's correlation method was used for calculating the correlation coefficient values and those significant with a *p* < 0.05 were considered significant. The analysis was performed using the *corrplot* v0.92 package (https://github.com/taiyun/corrplot) in R.

### 2.9 Phenome‐Wide Association Study

Phenome‐wide association studies (PheWAS) have emerged as a viable strategy to explore potential pleiotropic phenotypes associated with gene variants. Such studies are used to find associations between a genomic region of interest and phenotype traits measured in GWAS datasets. We used the online tool PheWAS (https://atlas.ctglab.nl/PheWAS) within GWASatlas database [56] to detect the genes with relevance to neurological pain in humans. A relevant association with a *p* < 0.05 was considered significant.

### 2.10 Functional Enrichment Analysis

The functional enrichment analysis of the module genes and DEGs was performed using WebGestalt (https://www.webgestalt.org/) [57] to identify significantly enriched Kyoto Encyclopaedia of Genes and Genomes (KEGG) pathways. For the enrichment analysis “protein coding genes” was selected as the reference set, whereas “minimum number of genes per category” was kept as 5. The *p*‐values were corrected using Benjamini‐Hochberg method [45] for multiple testing and the pathways with an FDR adjusted *p* ≤ 0.1 were considered significant. Furthermore, the module genes were submitted to Metascape tool (http://metascape.org) [58] to identify the enriched Gene Ontology biological processes (GO‐BPs) and explore their relationships in the form of a clustered network. The Metascape tool uses a heuristic algorithm to select the most informative terms from the GO clusters. It samples 20 top‐score clusters, selects up to the 10 best scoring terms (lowest p‐values) within each cluster, then connects all term pairs with Kappa similarity above 0.3.

### 2.11 Transcription Factor Analysis

Transcription factor (TF) enrichment analysis was performed using the Enrichr web tool (https://maayanlab.cloud/Enrichr) [59]. In Enrichr, “ChEA 2022” and “TRRUST Transcription Factors 2019” datasets were selected for the enrichment analysis. TFs enriched with a *p* < 0.05 were considered significant. TRRUST is a manually curated database containing human and mouse transcriptional regulatory networks that have been obtained from ~11,000 research articles, describing small‐scale experimental studies of transcriptional regulations [60]. Although the information in TRRUST is highly reliable, being a curated database, it is likely to suffer from low coverage. To compensate this, we used ChEA [61], which contains TF‐target data primarily from ChIP‐seq based high‐throughput studies from multiple sources including ENCODE, ReMap, individual publications and gene signatures resulting from single TF perturbations followed by genome‐wide gene expression experiments.

## 3 Results

### 3.1 WGCNA and Enrichment Analysis Identified the Pain Module

The co‐expression network was constructed using the gene expression data corresponding to 214 human DRG samples collected from the brain‐dead subjects. The normalized gene expression levels of the DRG samples were downloaded from the GEO dataset, GSE77968 and top 10,000 genes based on their mean expression were used for network construction using a soft thresholding power of 8, which was decided based on the scale‐free topology and mean connectivity (Figure 1A). As shown in Figure 1B, the top 10,000 genes were initially clustered into a total of 25 co‐expression modules, merging of which based on their eigengene values with a 75% similarity resulted in 13 modules, each containing a different number of genes (Figure 1C). The “brown” module had the most genes (*n* = 2173), whereas the “royalblue” module contained the fewest genes (*n* = 90). The “gray” module consisting of the unassigned genes was excluded from further analysis.

Furthermore, we performed the enrichment analysis using pain reference geneset for all 12 coexpression modules that were identified based on WGCNA analysis of DRG expression data. We intended to identify the coexpression module(s) that has significant over‐enrichment of the pain genes. The pain reference set contained a total of 455 genes that were collected and compiled from different publicly available resources, including RGD, GeneCards, DISEASES database and GWAS‐Catalog. The enrichment analysis revealed that blue module was the most significantly over‐enriched (enrichment ratio = 2.37; FDR *p* = 1.43E‐07) module (Figure 1D), and interestingly 51 of the 1181 genes in this module were pain reference genes. The only other module that was significant with an FDR *p* < 0.05 was “darkgreen” module; however, it was under‐enriched (enrichment ratio = 0.2) and contained only two pain reference genes. Thus, based on the enrichment analysis results, we decided to consider “blue” module as the “pain module” and used the genes in this module for further analysis.

### 3.2 Blue Pain Module Genes Are Significantly Involved in Brain and Nervous System‐Related Processes and Pathways

**FIGURE 2:**
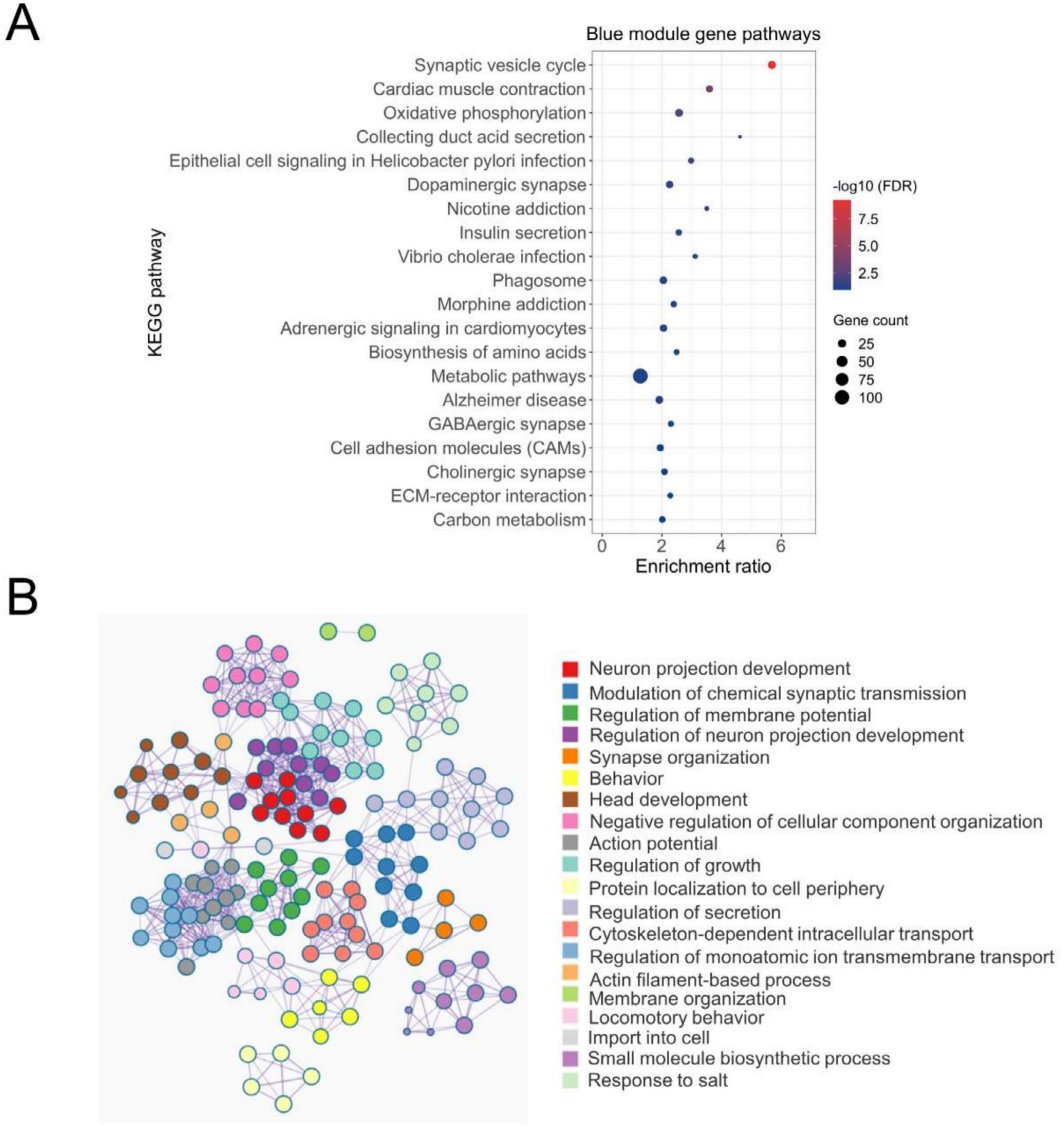
Functional analysis of blue pain module genes. (A) Bubble plot representing top 20 pathways significantly enriched by the blue pain module genes. Enrichment ratio = number of observed divided by the number of expected genes from each category. (B) The blue pain module genes were analyzed using the Metascape tool. The nodes represent enriched Gene Ontology biological process (GO‐BP) terms and are colored by their respective cluster IDs, whereas the edges link similar terms. The most significant term of the cluster is displayed as a label to represent that cluster.

To explore the functional importance of the blue pain module genes, we performed gene enrichment analysis using KEGG pathways and GO annotations. The analysis of the 1181 genes in the blue module found enrichment of various pathways and processes associated with brain/nervous system physiology and pathology (Figure 2A,B). As shown in Figure 2A, seven of the top 20 pathways were found to be directly linked to nervous system‐related physiology. “Synaptic vesicle cycle” was found to be the most significantly enriched KEGG pathway with 23 blue module genes (FDR *p* = 6.15E‐10). The other interesting pathways included “dopaminergic synapse” (*n* genes = 19; FDR *p* = 0.035), “morphine addiction” (*n* genes = 14; FDR *p* = 0.05), “GABAergic synapse” (*n* genes = 13; FDR *p* = 0.08), and “cholinergic synapse” (*n* genes = 15; FDR *p* = 0.09). Similarly, GO analysis showed enrichment of several nervous system‐related biological processes by the blue pain module genes. We also observed a close relationship between most of the GO‐BP annotations, indicating a significant overlap of genes among multiple GO‐BP annotations (Figure 2B). File S3 contains the list of significant KEGG pathways and GO‐BP annotations enriched by the blue module genes.

### 3.3 Identification of a Pain PPI Subnetwork Through the Analysis of Protein–Protein Interactions

**FIGURE 3:**
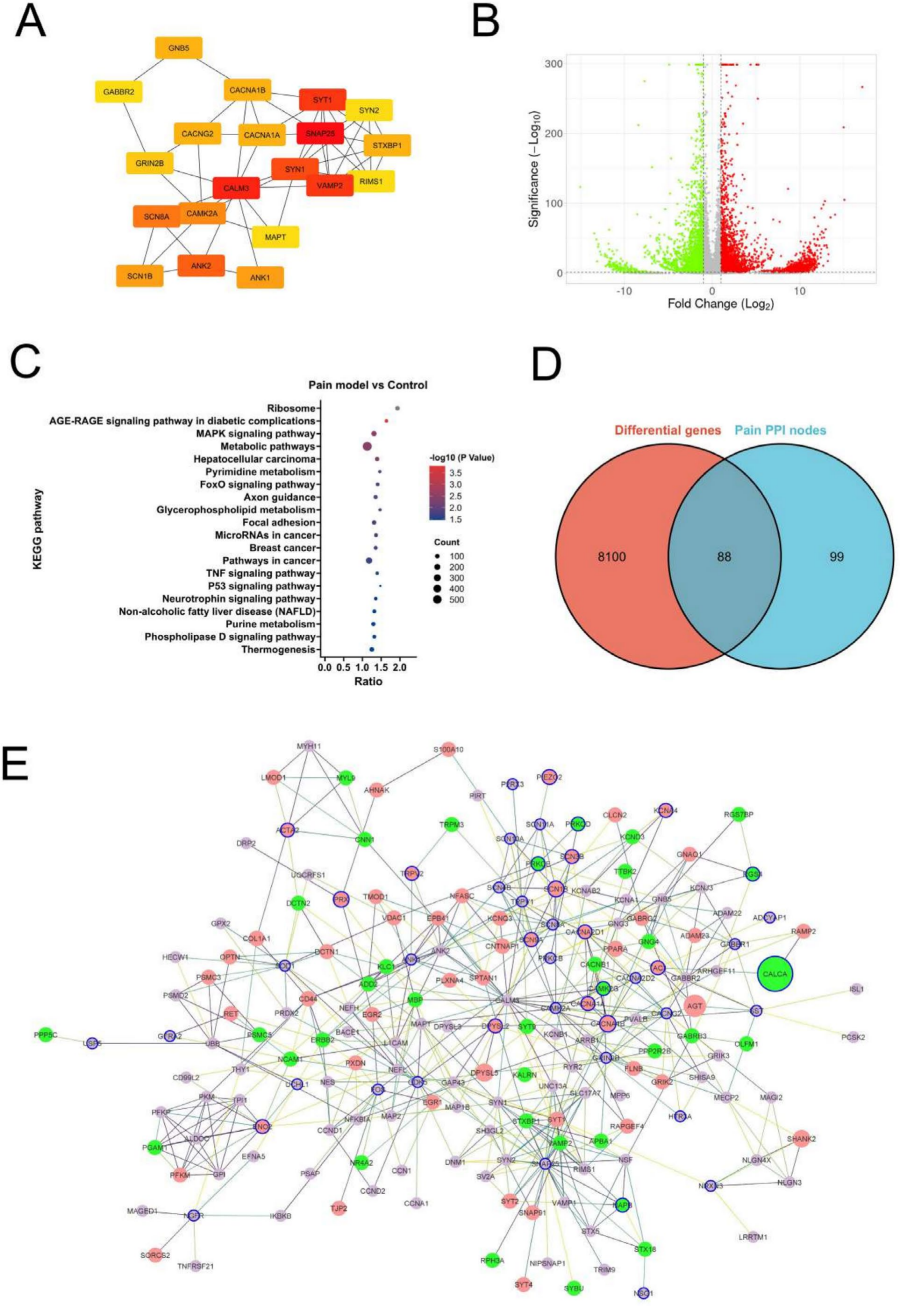
Identification of novel pain genes through PPI network of blue module and RNA sequencing of pain model and control mice. (A) Top 20 ranked proteins based on Cytohubba's Maximum Neighborhood Component algorithm. The color gradient of the nodes represents their ranks with dark red representing top rank and yellow representing bottom rank. (B) Volcano plot showing significantly differentially expressed genes between pain model and control mice. Red dots represent upregulated, green dots represent downregulated and gray dots represent genes with no change at a fold change threshold of 1.5 and FDR corrected *p* < 0.05. (C) KEGG pathway enrichment analysis of significantly differentially expressed genes between pain model and control mice. Top 20 pathways are shown. (D) Venn diagram showing count of overlapping genes between differentially expressed list and pain PPI subnetwork. (E) PPI network of pain reference genes with their direct interactors overlayed with differential expression information between pain model and control mice. Red nodes: upregulated genes; green nodes: downregulated genes; purple nodes: No significant change. The node size is directly proportional to the fold change of the genes. Nodes with blue border represent the pain reference genes. The edge color represents the score threshold with dark blue gradient representing higher scores and yellow gradient lines representing lower scores.

The gene/proteins involved in similar functions or processes tend to interact with each other. Therefore, we performed the protein‐interaction analysis of the blue pain module genes to identify important nodes (proteins). Among 1181 genes, 613 genes were found to be connected through 1521 edges (interactions) with a high threshold score of 0.7 based on the interaction information obtained from the STRING database (Figure S1). There were 46 pain reference genes that were part of this PPI network. *CALM3* was found to have highest degree with 38 interactions. To analyze the PPI network in the context of pain‐related genes, we extracted a subnetwork containing only the pain reference genes and their primary interactors (proteins directly interacting with the pain reference genes). The pain reference subnetwork contained a total of 187 nodes (including 46 pain reference genes and their 141 primary interactors) connected with 526 edges (Figure S2 and File S4). In this subnetwork, the pain reference gene *SNAP25* was found to have highest node degree with 29 interacting molecules followed by *CALM3* with 25 interactions. Furthermore, ranking of the nodes based on Cytohubba's Maximum Neighborhood Component (MNC) algorithm indicated SNAP25, CALM3 and vesicle‐associated membrane protein 2 (VAMP2) as the top 3 nodes in the pain PPI subnetwork (Figure 3A).

### 3.4 RNA Sequencing Between Pain Model and Control Mice Discovers Potential Novel Pain Genes

To further select the novel genes associated with pain, we performed RNA sequencing analysis of pain mouse model and compared the results with that of control mice. The RNA‐seq analysis between the pain model and control group resulted in a large number of significantly differentially expressed genes. A fold‐change of ≥ 1.5 and FDR corrected *p* < 0.05 resulted in 4751 upregulated genes and 3442 downregulated genes (Figure 3B and File S5) in the pain model compared to control mice. Top 20 up‐ or down‐regulated genes in pain model versus control mice are shown in Table 1. These genes were enriched for brain and nervous system‐related pathways, such as “axon guidance” (*n* genes = 88; FDR *p* = 0.0115), “cholinergic synapse” (*n* genes = 54; FDR *p* = 0.07), and “GABAergic synapse” (*n* genes = 43; FDR *p* = 0.08). Interestingly, the top 20 list contained several signaling‐related pathways, including “MAPK signaling pathway” (*n* genes = 143; FDR *p* = 0.003), “FoxO signaling pathway” (*n* genes = 69; FDR *p* = 0.011), “TNF signaling pathway” (*n* genes = 57; FDR *p* = 0.027), “Neurotrophin signaling pathway” (*n* genes = 61; FDR *p* = 0.034), and “Phospholipase D signaling pathway” (*n* genes = 72; FDR *p* = 0.034) (Figure 3C). Some of these pathways including “MAPK signaling”, “TNF signaling”, and “Neurotrophin signaling” have been implicated in neuropathic pain response.

**TABLE 1:** Top 20 up‐ or down‐regulated genes between pain model and control mice.

Gene symbol	Description	Log2 fold	FDR
*Nbdy*	Negative regulator of P‐body association	17.1	3.13E‐267
*Lcn2*	Lipocalin 2	15.1	9.58E‐106
*Slc24a5*	Solute carrier family 24, member 5	15.0	2.54E‐209
*Il18bp*	Interleukin 18 binding protein	14.0	1.45E‐84
*Afg1l*	AFG1 like ATPase	13.5	1.81E‐80
*Cstad*	CSA‐conditional, T cell activation‐dependent protein	13.2	8.75E‐28
*Gm20481*	Predicted gene 20,481	13.2	3.16E‐18
*Nts*	Neurotensin	13.1	2.56E‐37
*Rpp40*	Ribonuclease P 40 subunit	13.0	1.23E‐40
*Maob*	Monoamine oxidase B	12.9	1.83E‐63
*Pwwp3b*	PWWP domain containing 3B	12.8	6.80E‐104
*Zfp458*	Zinc finger protein 458	12.8	4.49E‐40
*Gm16867*	Predicted gene, 16,867	12.8	1.53E‐51
*Gm28042*	Predicted gene, 28,042	12.7	6.98E‐99
*Arntl*	Aryl hydrocarbon receptor nuclear translocator‐like	12.7	1.73E‐58
*H4c8*	H4 clustered histone 8	12.7	3.56E‐11
*Fkbp10*	FK506 binding protein 10	12.5	5.55E‐51
*Zfp773*	Zinc finger protein 773	12.5	3.58E‐60
*Uty*	Ubiquitously transcribed tetratricopeptide repeat containing, Y‐linked	12.5	2.09E‐93
*Lrriq3*	Leucine‐rich repeats and IQ motif containing 3	12.4	4.62E‐35
*Klk6*	Kallikrein‐related peptidase 6	−15.0	4.34E‐124
*Gm4767*	Predicted gene 4767	−13.4	9.34E‐58
*Ctsk*	Cathepsin K	−13.2	6.08E‐49
*Tyrobp*	TYRO protein tyrosine kinase binding protein	−13.1	9.11E‐18
*Ctss*	Cathepsin S	−13.0	1.21E‐38
*Ccnb2*	Cyclin B2	−13.0	3.58E‐42
*Moap1*	Modulator of apoptosis 1	−12.8	1.39E‐31
*C1qc*	Complement component 1, q subcomponent, C chain	−12.7	3.56E‐27
*Ada*	Adenosine deaminase	−12.2	4.71E‐28
*Hapln2*	Hyaluronan and proteoglycan link protein 2	−12.2	8.03E‐25
*Fcer1g*	Fc receptor, IgE, high affinity I, gamma polypeptide	−12.2	9.98E‐11
*Calca*	Calcitonin/calcitonin‐related polypeptide, alpha	−12.1	9.98E‐11
*Cyp2j12*	Cytochrome P450, family 2, subfamily j, polypeptide 12	−12.0	5.35E‐26
*Gsx2*	GS homeobox 2	−12.0	2.37E‐23
*Npm2*	Nucleophosmin/nucleoplasmin 2	−11.9	1.74E‐12
*Bub1*	BUB1, mitotic checkpoint serine/threonine kinase	−11.8	9.25E‐43
*Gins1*	GINS complex subunit 1 (Psf1 homolog)	−11.8	1.18E‐13
*C1qb*	Complement component 1, q subcomponent, beta polypeptide	−11.8	2.32E‐13
*Ribc1*	RIB43A domain with coiled‐coils 1	−11.7	9.11E‐18
*Melk*	Maternal embryonic leucine zipper kinase	−11.7	8.05E‐35

When we overlayed the differential expression information on the pain PPI network containing 187 nodes, we found that 88 genes in the network are common between both the sets (Figure 3D). Among these 88 genes, 20 were pain reference genes and the remaining were their direct interacting partners. The pain PPI network shown in Figure 3E displays the connections between these 88 genes, which not only shows their significant differential expression upon pain induction in mice but also their interaction at the protein level with each other at a high confidence. The significantly differentially expressed genes between pain model and control mice that directly interact with the “pain reference genes” can be considered as novel molecules with a higher importance to pain regulation and response. Therefore, we explored these genes further to establish their role as possible regulators of pain response.

### 3.5 Differentially Expressed Pain Network Genes Are Associated With Pain‐Related GWAS Traits

Next, we intended to explore, whether those 88 genes are associated with pain‐related GWAS traits through PheWAS analysis. Our analysis demonstrated that 53 of the 88 genes were associated with at least one neuropathic pain‐related phenotype in humans with *p* < 0.05 (File S6). *NCAM1*, *APBA1*, and *CAMK2G* were found to be associated with four traits each, whereas *CD44*, *KLC1*, *MYL9*, *RGS7BP*, *FLNB* and *TRPM3* were associated with three pain‐related treats each. Furthermore, with a higher significance threshold (*p* < 1E‐03), we found a total of 15 genes to be associated with neurological pain‐related traits (Table 2). Among these, *NCAM1* and *KALRN* were the top genes, each with two pain‐related GWAS traits. The most common traits were found to be “Neck or shoulder pain” and “knee pain” followed by “back pain” and “headache”. Although *NCAM1*, *SNAP91*, *APBA1*, and *KALRN* were associated with “Neck or shoulder pain”, *KCNQ3*, *SYT2*, *KALRN*, and *PIEZO2* were associated with “knee pain” (Table 2).

**TABLE 2:** Genes associated with human neurological pain‐related GWAS traits with *p* < 0.001.

Genes	Trait	*p*
*NCAM1*	Pain type(s) experienced in last month: Neck or shoulder pain	1.11E‐05
*NCAM1*	Pain type(s) experienced in last month: Back pain	0.000104
*ENO2*	Pain type(s) experienced in last month: Headache	0.000157
*SNAP91*	Pain type(s) experienced in last month: Neck or shoulder pain	4.64E‐05
*KCNQ3*	Pain type(s) experienced in last month: Knee pain	0.000467
*PSMC3*	Pain type(s) experienced in last month: Stomach or abdominal pain	0.000543
*SYT2*	Pain type(s) experienced in last month: Knee pain	0.000622
*GABRG2*	Pain type(s) experienced in last month: Hip pain	7.27E‐05
*MYL9*	Pain type(s) experienced in last month: Hip pain	2.04E‐05
*TJP2*	Pain type(s) experienced in last month: Headache	2.92E‐09
*PPP5C*	Pain type(s) experienced in last month: Back pain	0.000722
*APBA1*	Pain type(s) experienced in last month: Neck or shoulder pain	0.000155
*CAMK2G*	Pain type(s) experienced in last month: Back pain	4.22E‐05
*KALRN*	Pain type(s) experienced in last month: Neck or shoulder pain	1.56E‐05
*KALRN*	Pain type(s) experienced in last month: Knee pain	0.000306
*CALCA*	Pain type(s) experienced in last month: Headache	0.000304
*PIEZO2*	Pain type(s) experienced in last month: Knee pain	0.00015

### 3.6 Correlation of Potential Candidates With BXD Pain Phenotypes

**FIGURE 4:**
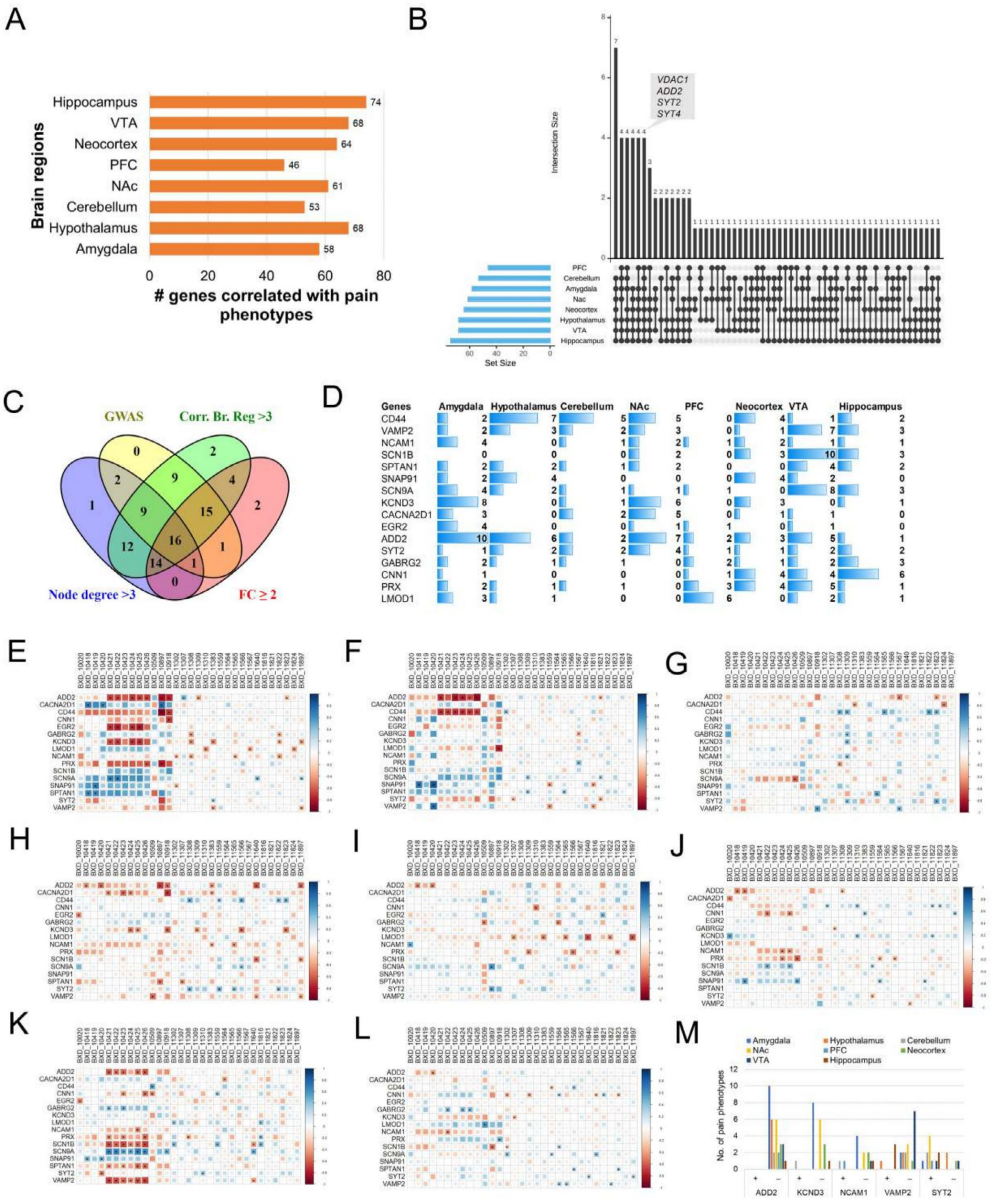
Correlation of gene expression from different brain regions with pain phenotypes in BXD mice and selection of top pain modulators. (A) Number of genes (of total 88 genes) correlated with at least one pain phenotype in each brain region of BXD mice. (B) Number of genes having significant correlations with pain phenotypes across multiple brain regions of BXD mice. (C) Selection of top genes associated with pain response/regulation based on PPI network, human GWAS traits, phenotype correlation in BXD mice and differential expression between pain model and control mice. A total of 16 genes were found to overlap across the four categories. (D) Expression of 16 genes from 8 brain regions of BXD mice is correlated with different number of pain phenotypes. (E–L) Correlation plots showing significant association of the expression of 16 pain response/modulator genes in amygdala (E), hypothalamus (F), cerebellum (G), NAc (H), PFC (I), neocortex (J), VTA (K), and hippocampus (L), respectively, with BXD pain phenotypes. The significant correlations are indicated with an asterisk. The square size and color intensity represents the relative correlation value. BXD identifiers represent pain phenotypes. (M) Number of positively (+) and negatively (−) correlated pain phenotypes with the expression of top 5 genes across different brain regions. The details of the pain phenotypes are found in File S2.

To further confirm the importance of these 88 genes in neuropathic pain, we used expression data from different brain regions of a well‐characterized murine GRP, the BXD mice. The expression levels from different brain regions of BXDs, including amygdala, hypothalamus, cerebellum, NAc, PFC, neocortex, and VTA were correlated with 31 neuropathic pain phenotypes in BXD mice. Both expression data and pain phenotype trait data have been generated by us or our collaborators and deposited in our GeneNetwork portal (http://genenetwork.org/). The brain regions that were considered for the correlation analysis have been known to be associated with pain response or regulation based on various human and mice studies [62]. The analysis suggested a significant correlation between most of the 88 genes and at least one pain phenotype (Figure 4A), although the top genes were different in different brain regions (File S7). For instance, the expression of 58 genes from amygdala was significantly correlated (*p* < 0.05) with at least one pain phenotype in BXD mice, and that 68 genes in hypothalamus were correlated with pain phenotypes. Similarly, the expression levels of 53 genes from cerebellum were correlated with the pain phenotypes. The top genes in amygdala were *Vdac1*, *Pgam1*, *Kcnd3*, and *Add2*, each correlated with at least 8 of the 31 BXD pain phenotypes considered, whereas the top 4 genes in hypothalamus were *Cd44*, *Vdac1*, *Add2*, and *Ttbk2*, each correlated with at least 6 pain phenotypes (File S7). Interestingly, the expression of *Vdac1*, *Add2*, *Syt2*, and *Syt4* in all 8 brain regions were significantly correlated with the pain phenotypes (Figure 4B).

### 3.7 *NCAM1* , *VAMP2* , *SYT2* , *ADD2* , and *KCND3* Were Identified as Top Genes for Pain Response or Regulation

Furthermore, we shortlisted 16 potential pain response or regulation‐related genes based on node degree in the PPI network, higher differential expression between pain model and control mice (fold change ≥ 2), pain‐related GWAS trait associations in humans, and correlation with BXD pain phenotypes across 8 different brain regions (Figure 4C). As shown in Figure 4D, the expression of these genes in 8 different brain regions correlated with varying number of pain phenotypes in BXD mice. Furthermore, 11 of these genes were found to be upregulated in the pain model compared to control mice, whereas the remaining were significantly downregulated. Among the 16 genes, four are already known to be pain‐response related (*SCN1B*, *SCN9A*, *CACNA2D1*, and *PRX*) based on our curated pain‐reference geneset, which indicates that the remaining 12 are potential novel pain response/regulator genes (Table 3). Among the potential novel genes, VAMP2 interacted with most proteins in the pain PPI network (*n* = 18). Its expression in seven different brain regions was significantly correlated with one or more pain phenotypes in BXD mice (Figure 4E–L). *NCAM1* was found to be associated with four neuropathic pain‐related GWAS traits in humans, two of which were highly significant with a *p* < 0.001 (Tables 2 and 3). Additionally, both these were found to be downregulated in pain model compared to control mice with a fold change of > 2. Furthermore, the expression of two genes, *ADD2* and *SYT2*, correlated with the pain phenotypes in all 8 brain regions of BXD mice. Both these genes interacted with 6 other proteins in the pain PPI subnetwork (Figure 3E). It is noteworthy that the mRNA expression of *SYT2* was found to be 100‐fold higher in the pain model than in control group based on our RNA sequencing results. Furthermore, *SYT2* was also found to be significantly associated with two neuropathic pain‐related GWAS traits. Interestingly, the gene‐pain phenotype correlations across all the 8 brain regions were found to be highest for *ADD2*, with maximum number of correlations in amygdala (Figure 4D and File S7). *KCND3* was another interesting gene, amygdala expression of which correlated with several pain phenotypes. When the number of positive and negative correlations is checked, *Add2*, *Kcnd3*, *Ncam1*, and *Vamp2* were found to be mostly negatively correlated with the pain phenotypes across all brain regions, whereas *Syt2* had higher number of positively correlated phenotypes (Figure 4M). This trend was in agreement with the differential expression, where *Syt2* was found to be upregulated, and the other 4 genes were downregulated in the pain model. Thus, our analysis suggested that these 5 genes, i.e., *NCAM1*, *VAMP2*, *SYT2*, *ADD2*, and *KCND3* could be potential novel genes for pain response or regulation. However, validation through knockin/knockout experiments may further strengthen the functional roles of these genes.

**TABLE 3:** Selected pain regulators by integrating protein interactions, GWAS, phenotype correlation, and differential expression data.

Gene symbol	Node degree	Pain reference gene	FDR	LogFC (Pain model vs control)	Correlated with pain phenotypes in following brain regions	GWAS count
*CD44*	8	—	4.94E‐02	6.49	Amy, Hyp, Cer, NAc, Neo, VTA, Hip	3
*VAMP2*	18	—	2.18E‐53	−1.37	Amy, Hyp, Cer, NAc, Neo, VTA, Hip	1
*NCAM1*	8	—	1.27E‐153	−1.22	Amy, Cer, NAc, PFC, Neo, VTA, Hip	4
*SCN1B*	12	Yes	6.97E‐03	8.75	NAc, Neo, VTA, Hip	1
*SPTAN1*	8	—	5.55E‐88	1.11	Amy, Hyp, Cer, NAc, VTA, Hip	1
*SNAP91*	4	—	6.77E‐46	1.2	Amy, Hyp, Neo, VTA	2
*SCN9A*	9	Yes	2.58E‐02	5.64	Amy, Hyp, Cer, NAc, PFC, VTA, Hip	1
*KCND3*	5	—	1.56E‐34	−1.12	Amy, Cer, NAc, Neo, Hip	2
*CACNA2D1*	8	Yes	1.63E‐27	1.19	Amy, Cer, NAc, Neo, VTA	2
*EGR2*	4	—	1.34E‐32	2.13	Amy, NAc, PFC, VTA	1
*ADD2*	6	—	3.06E‐18	−2.23	Amy, Hyp, Cer, NAc, PFC, Neo, VTA, Hip	1
*SYT2*	6	—	6.97E‐03	6.64	Amy, Hyp, Cer, NAc, PFC, Neo, VTA, Hip	2
*GABRG2*	7	—	8.95E‐17	1.61	Amy, Hyp, Cer, PFC, Neo, VTA, Hip	1
*CNN1*	5	—	3.92E‐11	−2.06	Amy, PFC, Neo, VTA, Hip	1
*PRX*	4	Yes	3.82E‐06	8.61	Amy, Hyp, Cer, PFC, Neo, VTA, Hip	1
*LMOD1*	4	—	1.43E‐09	1.32	Amy, Hyp, PFC, VTA, Hip	1

Abbreviations: Amy, Amygdala; Cer, Cerebellum; FC, Fold change; FDR, False discovery rate; Hip, Hippocampus; Hyp, Hypothalamus; NAc, Nuclear accumbens; Neo, Neocortex; PFC, Prefrontal cortex; VTA, Ventral tegmental area.

### 3.8 RUNX1 and MYCN Regulate the Pain Response Genes

**FIGURE 5:**
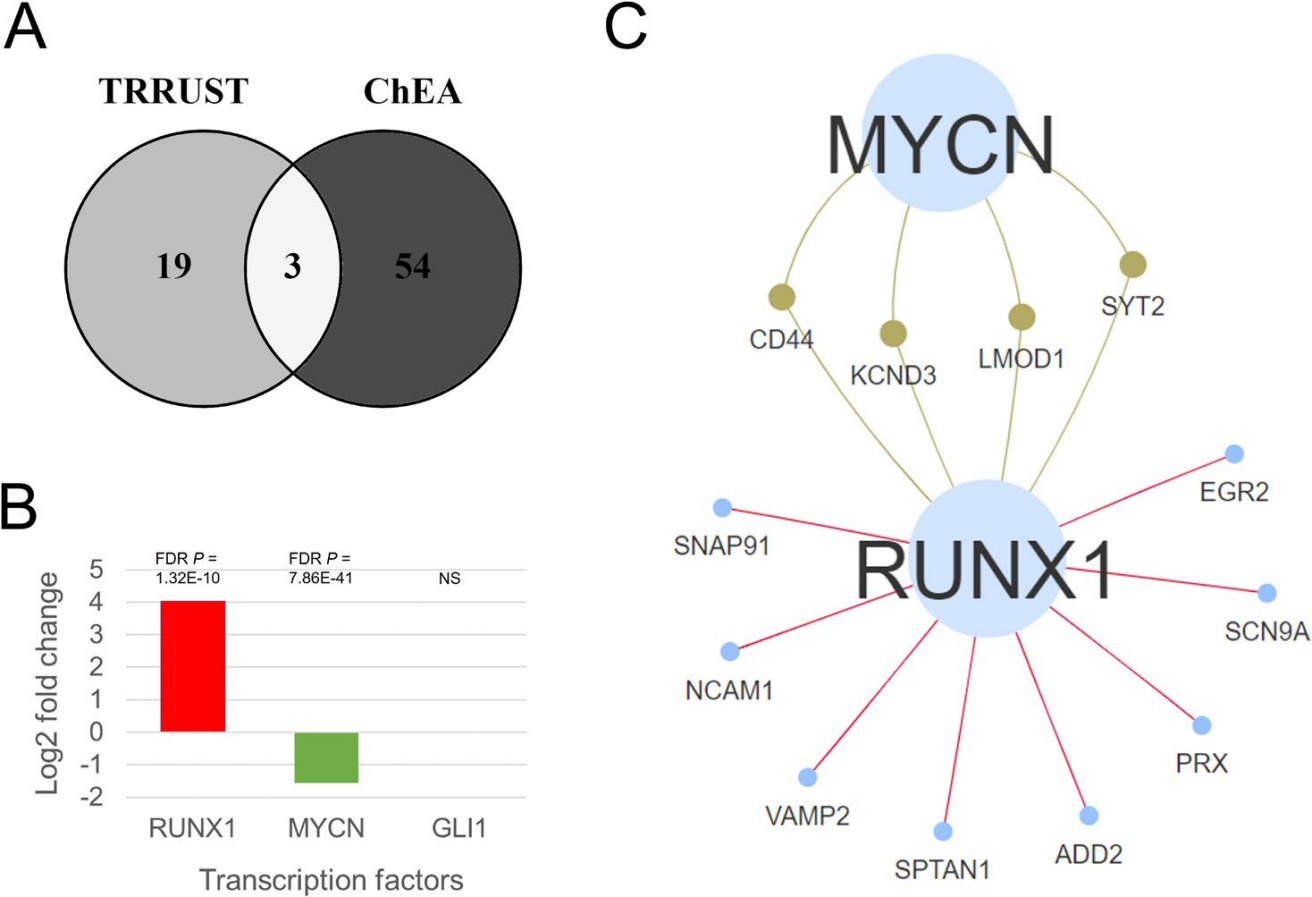
Transcription factors (TFs) targeting the novel pain genes. (A) Common TFs enriched by both TRRUST and ChEA databases. (B) Regulation of the common TFs in pain model compared to control mice. (C) Pain genes commonly targeted by both RUNX1 and MYCN. NS, Not significant.

To explore the regulation of the 16 pain response/regulation genes, we performed TF enrichment analysis using the curated and ChIP‐seq based TF‐target pairs. The results identified 3 TFs (GLI1, RUNX1 and MYCN) that were significantly enriched for the pain genes by both the tools (Figure 5A). Of these, *Runx1* and *Mycn* were found to be significantly different between pain model and control mice. Although *Runx1* was 16‐fold upregulated (log2FC = 4.02), *Mycn* was ~3‐fold downregulated (log2FC = −1.55) in the pain model (Figure 5B). The targets of RUNX1 included 12 of the 16 genes, whereas MYCN targeted 4 pain genes. All four genes targeted by MYCN were also targeted by RUNX1 (Figure 5C). Thus, the TF analysis suggests that RUNX1 and MYCN could be involved in the regulation of expression of these genes; however, their binding to the targets needs to be verified using experimental methods.

## 4 Discussion

Chronic pain is not a single disorder, but a complex presentation caused by various injuries or diseases, with diverse pathophysiological mechanisms [5, 63]. It has been well established that chronic pain is an expression of neural plasticity, both in the PNS as peripheral sensitization and in the CNS as central sensitization [64]. Both the DRG and the spinal cord dorsal horn (SDH) play key roles in the development of chronic pain, and they are involved in the transmission, regulation, and maintenance of pain signals through various molecular and cellular mechanisms [65]. Anatomical studies have confirmed that pain perception results from nociceptive signals produced by nociceptive neuron activation in the peripheral sensory nerve. Subsequently, the nociceptive signals are transduced via the DRG neurons, which synapse to SDH neurons and finally project to the thalamus and cerebral cortex [66]. We expect to screen for important molecules involved in both peripheral sensitization and central modulation of pain in chronic pain. Hence, there is a need to better understand the underlying pathways and identify targets or candidate genes for improved management and treatment of pain. To this end, in the current study, we used a cross‐species integrative approach to identify novel pain regulators or response genes. First, using human microarray profiling data from more than 200 DRG samples, we identified clusters of genes that had a similar expression pattern (co‐expressed gene modules) by using the widely used WGCNA method. The result of this analysis presented us with clusters/modules of genes behaving similarly, indicating they may be involved in similar functions or pathways. However, among these, there is a possibility that only one or a few specific modules may be harboring the pain genes. Therefore, to identify such a module, we performed an enrichment analysis for the module genes using pain‐related genes as a reference (pain reference genes) that were collected from multiple gene databases/resources corresponding to human and mice. Among the 12 coexpression gene modules identified, only two modules, blue and darkgeen were found to be significantly enriched for the pain genes. Only the blue module was found to be over‐enriched and harbored 51 pain reference genes. Hence, we considered this module as the “pain coexpression module” for further analysis. A similar enrichment approach has been successfully used by us in our previous studies to identify candidate genes associated with glaucoma [67] and intraocular pressure [68]. Furthermore, a recent study by Wistrom et al., compiled a set of 242 high confidence genes linked to pain‐associated behavior in genetic models from literature spanning over three decades [1]. We used this set of 242 genes to further validate our blue module by comparing the module genes (*n* = 1181) with the set of genes in the literature and as expected, the blue module was again found to be over‐enriched with a better threshold this time (no. of overlapping genes: 41; enrichment ratio = 3.57; FDR *p* = 1.571E‐11). Thus, based on these enrichment results using two different pain reference sets, the blue module was considered as pain module and was used for further exploration.

To explore the functional significance of the blue pain module, we subjected the genes to GO and pathway enrichment analysis. The results demonstrated significant enrichment of various biological processes and pathways related to brain and nervous system. Furthermore, the network of GO‐BPs demonstrated a tight interaction between several processes that were related to neuron development, synapse organization, and synaptic transmission. The most significantly enriched KEGG pathway was “synaptic vesicle cycle”. The synaptic neurons and vesicles play an important role in pain response [69]. The synaptic vesicles are involved in the chemical events that are used to transmit the impulse between neurons. With the help of the N‐ethylmaleimide‐sensitive‐factor activating protein receptor (SNARE) proteins, the synaptic vesicles fuse to the presynaptic axon terminal membrane in the neurons, resulting in immediate release of neurotransmitters and calcium ions into the synaptic cleft via exocytosis. The released neurotransmitters then diffuse and bind to their cognate receptors located on the adjacent postsynaptic neuron, causing a localized action potential [69]. The other interesting pathways in the top 20 list were dopaminergic synapse, GABAergic synapse, and cholinergic synapse‐related pathways. There is accumulating evidence that dopamine systems in the brain are involved in the modulation of chronic pain [70, 71], although their primary role as a neurotransmitter is to mediate reward and motivation. A review by Li et al. [72] discusses in detail the role of descending dopaminergic pathways in the regulation of different types of pain. GABAergic neurons make over 20% of all brain neurons and this is a major inhibitory system in the brain. GABA (gamma‐aminobutyric acid), the main neurotransmitter of GABAergic system binds to the receptors on inhibitory neurons, leading to a decrease in neural activity. Given the importance of GABAergic system, its disruption can lead to various diseases, including chronic pain and depression [73, 74]. A study by Sullere et al. [75] highlights the novel cholinergic circuitry and receptor mechanisms that are involved in relieving pain despite opioid tolerance without any evidence of the development of analgesic tolerance, or withdrawal symptoms, stressing the potential clinical relevance of the cholinergic circuitry.

We performed PPI network analysis of the blue pain module genes to explore the relationships among them and eventually select the proteins with high connectivity to pain specific molecules. To further focus on the pain‐related network, we extracted a subnetwork containing only pain proteins and their primary interactors. The pain PPI network contained 187 nodes with 46 pain reference genes/proteins and 141 interactors with SNAP25 as with the highest node degree, closely followed by CALM3 with 25 interactions. Furthermore, the top 3 nodes based on their importance in the network as suggested by MNC algorithm were SNAP25, CALM3, and VAMP2. Synaptosome‐associated protein 25 (SNAP25), a component of the SNARE complex, is a presynaptic plasma membrane protein involved in the regulation of neurotransmitter release and has been implicated in neuropathic pain [76, 77, 78] and neuropsychiatric disorders [79, 80]. Calmodulin 3 encoded by *CALM3* gene, is a member of a family of proteins that binds to calcium and functions as an enzymatic co‐factor. Hasan et al. [81] have shown that *CALM3* is responsible for calcium‐dependent regulation of TRPA1 ion channels, which is known to be involved in many sensory disorders, such as pain, itch, and neuropathy. Furthermore, the expression of *CALM3* has been shown to increase by morphine through the stimulation of μ‐opioid receptor in rat cells [82]. VAMP2, another key component of SNARE functions by forming a stable complex with SNAP25 and syntaxins [83, 84]. A study by Salpietro et al. [85] identified two single‐amino‐acid deletions and three non‐synonymous variants that affected the conserved residues within the C terminus of the VAMP2 SNARE motif. Furthermore, variants in *SNAP25* and *VAMP2* that affect their expression have been implicated in migraine [86].

To identify the differentially expressed pain PPI network proteins, we performed RNA‐seq between pain model and control mice. The analysis resulted in ~8000 genes to be significantly different between pain model and control. *Nbdy* (negative regulator of P‐body association) was found to be the top upregulated gene followed by *Lcn2* (lipocalin 2) and *Slc24a5* (solute carrier family 24, member 5), whereas *Klk6* (Kallikrein‐related peptidase 6) was the top downregulated gene followed by *Gm4767* and *Ctsk* (cathepsin K). *Nbdy* involved in negative regulation of cytoplasmic mRNA processing, is relatively a new gene with a very few studies and has been implicated in pancreatic cancer [87]. However, its role in pain response/regulation is yet to be studied. The next best upregulated gene *Lcn2*, however was found to be associated with pain. Its deficiency reduced pain hypersensitivity, microglial activation, and chemokine production and as suggested by Jeon et al., it can be a target for the treatment of neuropathic pain [88]. *Klk6*, a member of the family of secreted serine proteinases has been identified as an important player in different cancers and in inflammation response. Oikonomopoulou et al. [89] demonstrated that *KLK6* may play a role in regulating the inflammatory response and pain perception by activating/inactivating a group of proteinase‐activated receptors. When we overlayed the differential expression information on the pain PPI network, 88 genes were found to be common. GWAS analysis of these genes through PheWAS, *NCAM1* and *KALRN* were found to be particularly interesting. Neural cell adhesion molecule 1 (*NCAM1*) is a member of the immunoglobulin superfamily and plays a key role in the development of the nervous system by regulating cell migration, and neurogenesis. Ko et al. [90] demonstrated that peripheral nerve injury induced increase in the turnover of anterior cingulate cortical *NCAM1* mediates synaptic reorganization and contributes to the behavioral sensitization, indicating its potential for neuropathic pain treatment. *NCAM1* has also been implicated in neurological disorders, such as autism and possibly other neuroinflammation‐related diseases [91]. Kalirin RhoGEF kinase (KALRN) encodes a protein that interacts with the huntingtin‐associated protein 1, a vesicle trafficking protein. This gene has been regarded as a central regulator of synaptic function and plays a key role in synaptic plasticity [92]. Mutations in *KALRN* have been shown to be associated particularly with neuronal diseases due to aberrant synapse formation [92]. Furthermore, a study by Parnell et al. indicated that reduced kinase activity of KALRN owing to E1577K mutation impairs neuroarchitecture and likely contributes to impaired neurodevelopment [93]. Given its importance in neurophysiology, this gene may be involved in pain response or regulation; however, further studies are required to delineate the exact mechanism.

Next, we correlated the mRNA expression of 88 genes from different brain regions that are known to directly or indirectly take part in pain response or regulation with pain phenotypes collected by us and our collaborators using the largest mouse GRP, the BXD strains. The expression from the following brain regions were considered for the correlation analysis: amygdala, hypothalamus, cerebellum, NAc, PFC, neocortex, VTA, and hippocampus due to their involvement in pain [62, 94, 95]. The analysis suggested different number of genes that were correlated with different phenotypes across the 8 brain regions. It is noteworthy that the following four genes: *Vdac1*, *Add2*, *Syt2*, and *Syt4* were correlated with the pain phenotypes in all 8 brain regions. *Syt* group of genes encode synaptic vesicle membrane proteins and are involved in neurotransmission, synaptic plasticity, and vesicle trafficking [96, 97]. Although *Vdac1* (v*oltage dependent anion channel 1*) is a voltage‐dependent anion channel and a major component of the outer mitochondrial membrane, *Add2* (*adducin 2*) is a membrane‐cytoskeleton‐associated protein. ADD2 is a heterodimeric protein and is composed of different subunits, with alpha and gamma being ubiquitously expressed, whereas adducin beta expression is restricted to brain and hematopoietic tissues. Although our results showed a significant correlation for both these genes with pain phenotypes, their exact role in pain response and regulation is yet to be explored.

Finally, based on multiple criteria including PPI node statistics, higher differential expression between pain model and control mice, pain‐related GWAS trait associations in humans, and correlation with BXD pain phenotypes across different brain regions, we narrowed down from a list of 88 potential pain genes to 16 genes that may have strong associations with pain response and regulation. Among these, 4 are already part of the pain reference list, leaving 12 as novel candidates. Of these, *NCAM1*, *VAMP2*, *SYT2*, *ADD2*, and *KCND3* were prioritized. Furthermore, when we looked at the regulation of these 16 pain‐related genes, GLI1, RUNX1, and MYCN emerged as the key TFs regulating these genes. Interestingly, RUNX1 and MYCN were also significantly differentially expressed between pain model and control mice strengthening our confidence in these regulators. Although RUNX1 (Runt‐related transcription factor 1) was upregulated, MYCN (MYCN proto‐oncogene, BHLH transcription factor) was significantly downregulated in the pain model compared to control.

## 5 Conclusion

In the current study, we used a cross‐species integrated omics approach to identify strongly associated candidate genes related to pain response or regulation. The five genes that were shortlisted (*NCAM1*, *VAMP2*, *SYT2*, *ADD2*, and *KCND3*) show correlation with pain phenotypes in BXD mice, differential expression in pain model versus control and significant human GWAS associations with pain traits. Although some of these have been implicated in pain physiology, others are yet to be explored and provide an opportunity to study these novel pain candidates. Thus, our study identifies novel candidates related to pain response or regulation; however, interventional validations are warranted to confirm their underlying mechanisms.

## Author Contributions

Ying Chen: investigation, writing original draft preparation, writing – reviewing and editing. Akhilesh K. Bajpai: data curation, visualization, investigation, writing original draft preparation. Qingqing Gu: validation, investigation, writing – reviewing and editing. Junpu Ruan: validation, writing – reviewing and editing. Ran Zhang: writing reviewing and editing. Gang Chen and Lu Lu: conceptualization, writing – reviewing and editing, supervision, funding acquisition.

## Conflicts of Interest

The authors declare no conflicts of interest.

## Supporting information


File S1.

File S2.

File S3.

File S4.

File S5.

File S6.

File S7.

Figure S1.

Figure S2.

## Data Availability

The data that support the findings of this study are available from the corresponding author upon reasonable request.
