# Supplementary material for: Discovery of Novel Pain Regulators Through Integration of Cross‐Species High‐Throughput Data
Source: CNS Neurosci Ther. 2025 Feb 9;31(2):e70255. doi: 10.1111/cns.70255 (PMC11807727; doi:10.1111/cns.70255)
Supplement: Supplementary file 8 — Figure S1. [file CNS-31-e70255-s007.pdf]

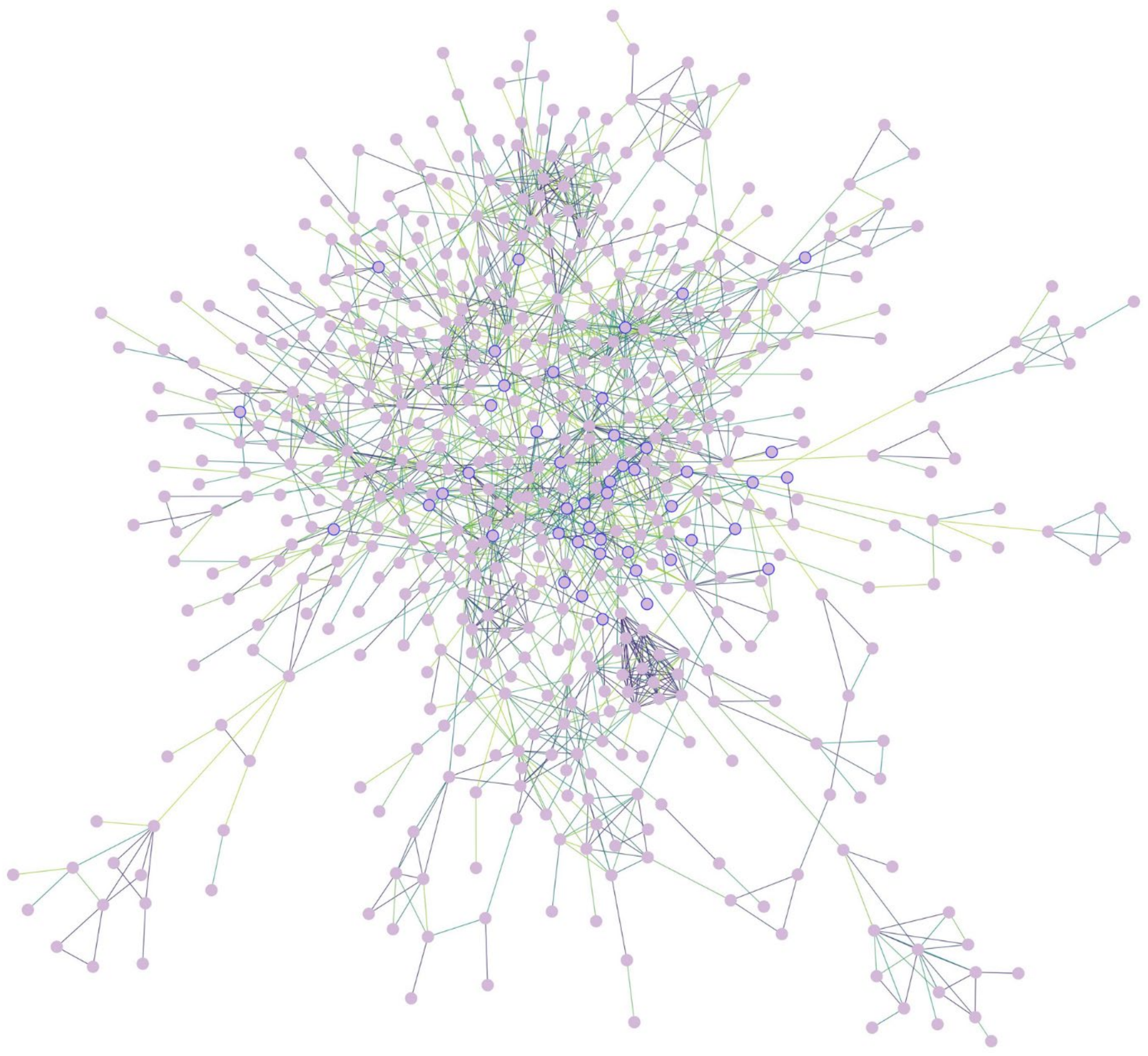

Supplementary Figure 1. PPI network of blue pain module genes. Nodes represent proteins and edges represent interactions between them. Nodes with blue border: Pain reference genes based on data collected from multiple publicly available sources. The edge color represents the score threshold with dark blue gradient representing higher score and yellow gradient lines representing lower score.
