# Supplementary material for: Discovery of Novel Pain Regulators Through Integration of Cross‐Species High‐Throughput Data
Source: CNS Neurosci Ther. 2025 Feb 9;31(2):e70255. doi: 10.1111/cns.70255 (PMC11807727; doi:10.1111/cns.70255)
Supplement: Supplementary file 9 — Figure S2. [file CNS-31-e70255-s003.pdf]

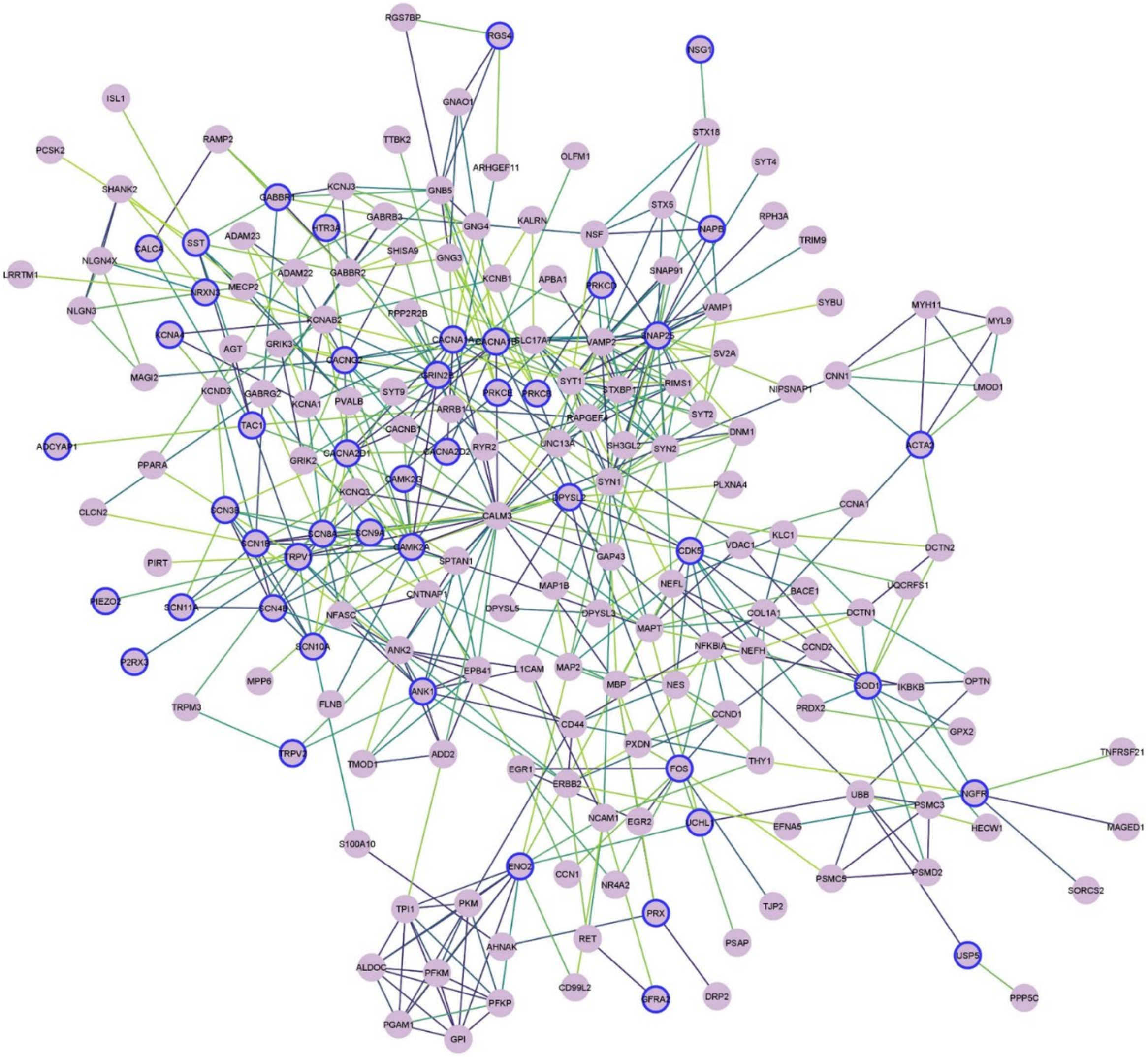

Supplementary Figure 2. A PPI subnetwork of pain reference genes and their direct interactors (also referred to as "pain PPI subnetwork"). Nodes represent proteins and edges represent interactions between them. Nodes with blue border: Pain reference genes based on data collected from multiple publicly available sources. The edge color represents the score threshold with dark blue gradient representing higher score and yellow gradient lines representing lower score.
